# Genomic Organization, Phylogenetic Comparison, and Differential Expression of the Nuclear Factor-Y Gene Family in Apple (*Malus Domestica*)

**DOI:** 10.3390/plants10010016

**Published:** 2020-12-24

**Authors:** Yanjie Qu, Yaping Wang, Jun Zhu, Yugang Zhang, Hongmin Hou

**Affiliations:** 1 College of Horticulture, Qingdao Agricultural University, Qingdao 266109, Shandong, China; quyanjieqau@gmail.com (Y.Q.); yapingwang113@gmail.com (Y.W.); junzhu@qau.edu.cn (J.Z.); ygzhang@qau.edu.cn (Y.Z.); 2 Qingdao Key Laboratory of Genetic Development and Breeding in Horticultural Plants, Qingdao Agricultural University, Qingdao 266109, Shandong, China

**Keywords:** *NF-Y* genes, evolution, abiotic stress, synteny analysis

## Abstract

The nuclear factor Y (NF-Y) as a transcription factor plays an important role in plants growth and development, and response to stress. However, few genome-wide analyzes and functional research of the NF-Y family has been undertaken in apple (*Malus domestica* Borkh.) so far. In this study, we comprehensively identified the 43 *MdNF-Y* genes in apple, which dispersedly distributed among the three subgroups based on their sequence alignment analysis, including 11 *MdNF-YAs*, 22 *MdNF-YBs* and 10 *MdNF-YCs*. The members in the same subgroups had similar evolution relationships, gene structures, and conserved motifs. The gene duplication analysis suggested that all the genes were dispersed followed by 27 segmental duplication. Moreover, based on synteny analysis of *MdNF-Y*s with eight plant species results suggested that some ortholog genes were preserved during the evolution of these species. Cis-element analysis showed potential functions of *MdNF*-*Ys* in apple growth and development and responded to abiotic stress. Furthermore, the interaction among MdNF-Ys protein were investigated in yeast two-hybrid assays. The expression patterns of *MdNF*-*Ys* in tissue-specific response reveled divergence and might play important role in apple growth and development. Subsequently, whole *MdNF-Y* genes family was carried out for RT-PCR in response to five abiotic stress (ABA, drought, heat, cold, and salinity) to identify their expression patterns. Taken together, our study will provide a foundation for the further study to the molecular mechanism of apple in growing development and response to abiotic stresses.

## 1. Introduction

Transcription factors (TFs) control the transcription or expression of downstream target genes by interacting with cis-elements through covalent binding to the DNA binding domain. Nuclear factor Y (NF-Y) TFs, known as CCAAT-binding factors (CBFs) or heme activator proteins (HAPs), play a critical regulatory role in plant vital movement by binding to the CCAAT element. NF-Y consists of three distinct subunits including NF-YA (also known as CBF-B/HAP2), NF-YB (CBF-A/HAP3), and NF-YC (CBF-C/HAP5) [1]. NF-Y is ubiquitously expressed in most eukaryotes and in mammals and yeast, each subunit is encoded by only one or two *NF-Y* genes. However, in plants, each NF-Y subunit has evolutionarily formed relatively large gene families expressed from multiple *NF-Y* genes. For example, in the model plant *Arabidopsis thaliana,* a total of 30 *AtNF*-*Y* genes exist including 10 *AtNF*-*YAs*, 10 *AtNF*-*YBs,* and 10 *AtNF*-*YCs* [2,3]. In addition, the three subunits each possess conserved DNA-binding domains and mutual interaction domains to form heterotrimeric complexes. It is also worth noting that NF-YA and NF-YC family members have a nuclear localization signal (NLS), which NF-YB members generally lack [2]. The NF-YB proteins are first translocated from the cytoplasm to the nucleus as part of a dimer complex with NF-YC. Subsequently, the dimer complex combines with NF-YA to form a mature heterotrimeric NF-Y complex transcription factor that binds to CCAAT boxes in the promoters of target genes [4,5]. Moreover, some of the NF-Y subunits also interact with other proteins besides the NF-YA subunit to regulate plant growth. For instance, *AtNF-YB3* and *AtNF-YC2* interact with post-proteolytic bZIP28 and *AtNF-YA4,* respectively, to assemble a complex that regulates the response to endoplasmic reticulum (ER) stress in *Arabidopsis* [6]. *AtNF*-*YC9* is a positive regulator involved in abscisic acid (ABA) signaling by interacting with ABA-responsive bZIP transcription factor ABA-INSENSITIVE5(ABI5) [7,8]. The *AtNF-YC4* gene interacts with the Qua-Quine Starch (QQS) orphan gene to regulate carbon and nitrogen allocation and reduce susceptibility to pathogens and pests [9,10].

Since the discovery of the NF-Y family in *Arabidopsis*, orthologous genes have been isolated and identified at genome-wide levels from other crops, including but not limited to walnut (*Juglans regia L.*) (17 *JrNF-YAs*, 9 *JrNF*-*YBs*, and 7 *JrNF*-*YCs*) [11], citrus (*Citrus sinensis*) (6 *CsNF*-*YAs*, 11 *CsNF*-*YBs*, and 5 *CsNF*-*YCs*) [12], chickpea (*Cicer arietinum L.*) (8 *CaNF*-*YAs*, 21 *CaNF*-*YBs*, and 11 *CaNF*-*YCs*) [13], castor bean (*Ricinus communis*) (6 *RcNF*-*YAs*, 12 *RcNF*-*YBs*, and 7 *RcNF*-*YCs*) [14], grape (*Vitis vinifera L.*) (8 *VvNF*-*YAs*, 18 *VvNF*-*YBs*, and 8 *VvNF*-*YCs*) [15], and peach (*Prunus persica L.*) (6 *PpNF*-*YAs*, 12 *PpNF*-*YBs*, and 6 *PpNF*-*YCs*) [16]. Many reports have demonstrated that these *NF-Y* genes play various vital functions in plant development and stress resistance, including seed germination [17], endosperm development [18], flowering time [19,20], photosynthesis [21], root nodule formation [10,22], hormone response [23], ER stress response, and abiotic and biotic stress response [6,24].

Abiotic stresses, such as osmotic stresses (drought or salinity) and temperature stresses (cold or heat), are increasingly becoming environmental factors that are limiting fruit productivity and quality worldwide. Previous reports have revealed that the *NF-Y* genes are involved in regulating abiotic stress via ABA-dependent or independent pathways. In *Arabidopsis*, overexpression of *AtNF*-*YA5*, *AtNF*-*YB1,* or *AtNF-YB2* has been shown to enhance drought stress tolerance by regulating the expression of stress-responsive genes [25,26,27]. Similarly, overexpression of *OsNF*-*YA7* has been shown to improve drought tolerance of transgenic rice, via an ABA-independent pathway [24]. The heterologous overexpression of *CdtNF*-*YC1* from hybrid bermudagrass improved transgenic rice tolerance to drought and salt stress [28]. Additionally, *ZmNF*-*YB2* was demonstrated to have a significant role in transgenic maize drought stress tolerance [27,29]. Furthermore, *ZmNF*-*YA3* also elevated transgenic plant tolerance to heat and drought stress by binding to *ZmMYC4, ZmbHLH92, and ZmFAMA* in the promoter region [29]. Overexpression of *PdNF-YB21* increased the drought resistance of transgenic poplars by positively regulating root growth and enlarging xylem vessels [30]. Recently, some studies have demonstrated that miRNA responded to abiotic stresses by regulating the expression of their target gene *NF-Y* [31]. The overexpression of soybean miR169c in transgenic *Arabidopsis* conferred increased drought stress sensitivity via inhibiting the expression of its target *AtNF*-*YA* gene [32]. The expression of most of *Zma-miR169* genes and their target *ZmNF-YA* genes were diverse changes during drought, salinity and ABA treatments [33,34].

As one of the most important economic crops, apples are widely cultivated in the temperate regions of the world. Unfortunately, with global climate change, apple trees also face many abiotic stresses during their growth and development. However, the role of *NF-Y* genes in stress tolerance in apple trees remains elusive. Based on the complete apple (*Malus domestica* Borkh.) genome released in 2010, the possibility of investigating the *MdNF*-*Y* gene family was offered in the species [35]. In this work, we analyzed the members of the apple *NF-Y* gene family (*MdNF*-*Y*) and determined their chromosomal location and detailed genetic information. Further, we characterized the apple NF-Y protein sequences, including the construction of a phylogenetic tree, detection of conserved motifs, and gene structure and synteny analysis. Then, to assess the function of *MdNF-Y* genes, we analyzed the cis-regulatory elements in the promoters and their transcriptional expression in different tissues. Yeast two-hybrid (Y2H) assays were conducted to study the interaction between various MdNF-Y subunits. The transcription profiles of the *MdNF-Y* genes were detected under various abiotic stresses. Our results provide a foundation for further study of the functional and regulatory mechanisms controlled by the *MdNF-Y* gene family.

## 2. Results

### 2.1. Identification and Characterization of NF-Y Family Genes in Apple

Following the removal of redundant sequences, we initially identified 11 *MdNF*-*YAs*, 26 *MdNF*-*YBs*, and 12 *MdNF*-*YCs* through Hidden Markov Model (HMM) analysis of the *M. domestica* genome (https://www.rosaceae.org/). Four putative *MdNF*-*YBs* (MD03G1280100, MD06G1209300, MD11G1164600, and MD14G1219800) and two putative *MdNF*-*YCs* (MD02G1273400 and MD07G1042300) did not contain the core structure of the NF-Y domain, so we removed them from further analysis. In order to facilitate further research, these genes were renamed according to their locations in chromosomes and the *Arabidopsis* nomenclature [2,36] (Table 1). Finally, the MdNF-YA, MdNF-YB, and MdNF-YC subunit families contained 11 (*MdNF*-*YA1*- *A11*), 22 (*MdNF*-*YB1*-*YB22*), and 10 (*MdNF*-*YC1* -*YC10*) members, respectively.

The gene length, coding sequence (CDS), and protein sequence of *MdNF*-*Y*s ranged from 365 bp (*MdNF-YC3*) to 9824 bp (*MdNF-YB17*), from 207 bp (*MdNF-YA4*) to 1077 bp (*MdNF-YA3*), and from 68 (*MdNF-YA4*) to 457 (*MdNF*-*YB19*) amino acids (aa), respectively. The isoelectric points (pI), and the molecular weights (MW) of MdNF-Y proteins varied from 5.15 (MdNF-YC4) to 9.63 (MdNF-YA10), and from 7449.24 Da (MdNF-YA4) to 38,855.31 Da (MdNF-YA3), respectively. The detailed information of the *MdNF-Y* genes including protein and CDS lengths is shown in Table S1.

**Figure 1:**
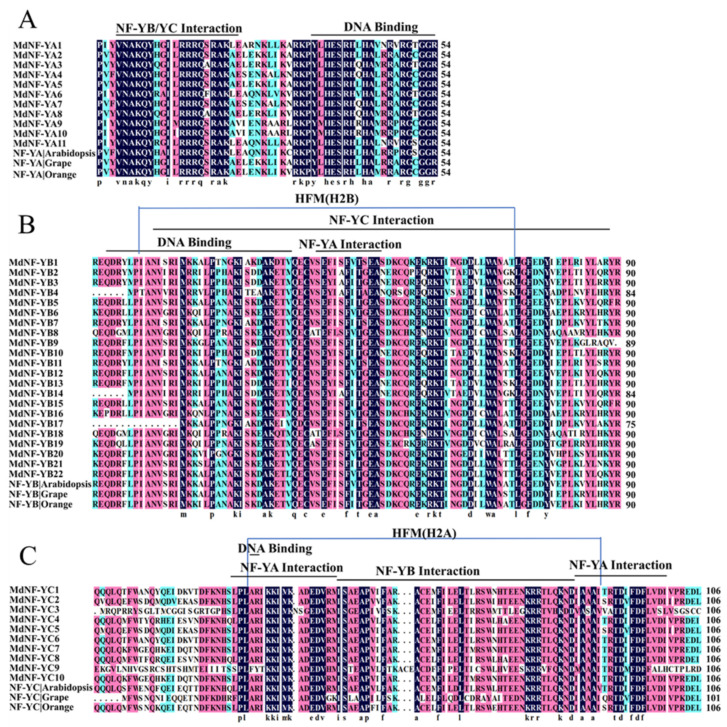
Multiple sequences alignment of the conserved domains between MdNF-Y (*M. domestica*), NF-Y Arabidopsis (*A. thaliana*) and NF-Y Grape (*V. vinifera*). The level of these three species amino acid homology = 100%, ≥75% and ≥50% are colored by blue, cyan, and pink boxes, respectively. (**A**) MdNF-YA subfamily; (**B**) MdNF-YB subfamily; (**C**) MdNF-YC subfamily. Multiple sequences alignment was constructed by DNAMAN software.

In addition, the three NF-Y subunits contained highly conserved domains among the previously reported species such as Arabidopsis (*A. thaliana*), grape (*V. vinifera*), and orange (*C. sinensis*) [2,12,15]. Similarly, multiple alignments indicated that apple NF-Y subunits also contained the same highly conserved regions as Arabidopsis, grape, and orange NF-Y subunits (Figure 1). The conserved central domain of the MdNF-YA, MdNF-YB, and MdNF-YC subunits, respectively, consisted of about 54, 90, and 81 aas, and three *MdNF*-*Y* subunits all contained a DNA binding domain. Moreover, the MdNF-YA contained an NF-YB/C interaction domain, and the MdNF-YB and MdNF-YC both contained an NF-YA interaction domain (Figure 1). These domains were necessary for NF-YA, NF-YB, and NF-YC to form a heterotrimeric complex to bind to CCAAT boxes [37]. Furthermore, the MdNF-YB and MdNF-YC both contained a histone-fold motif (HFM) resembling the core of H2B and H2A, respectively.

### 2.2. Phylogenetic Tree, Conserved Motifs, and Gene Structure Analysis of MdNF-Y Family

**Figure 2:**
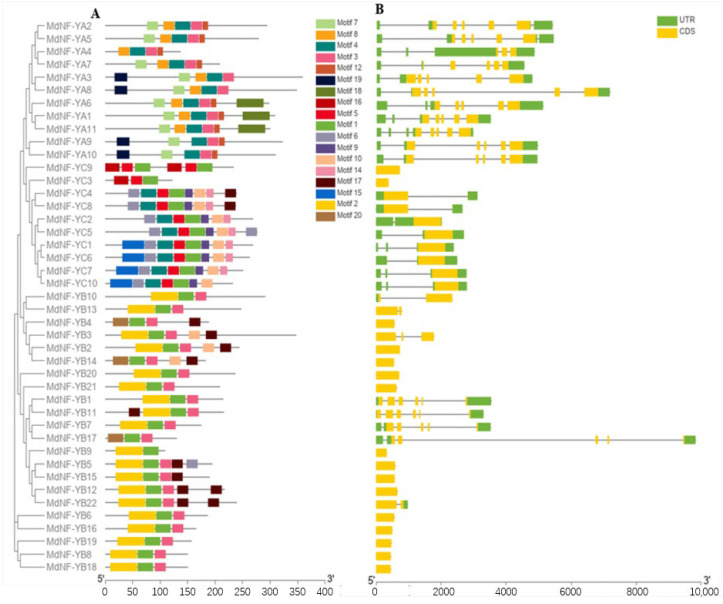
Conserved motifs (**A**) and gene structure (**B**) analysis in each *MdNF*-*Y* subfamily. Different colors of figure A represent the type of motifs *MdNF*-*Y* genes have. The green and yellow color box of figure B respectively indicates the coding sequences (CDS) and untranslated regions (UTR). Intron are shown with black lines. Scale bars located on the bottom side of figure representing the relative position based on the kilobase scale.

To deduce the potential evolutionary diversity and relationship of MdNF-Y subunit genes, we constructed a phylogenetic tree and analyzed the conserved motifs and gene structure for each MdNF-Y subunit (Figure 2). For each MdNF-Y subunit the corresponding genes had close phylogenetic relationships and consistently displayed conserved motifs and gene structures. For example, *MdNF*-*YA9* and *MdNF*-*YA10* were closely related evolutionarily and they presented similar motifs type (motif 3, 4, 7, 12, and 19) (Figure 2A). On the other hand, differences between various MdNF-Y subunits were evident in terms of major motifs. For example, motifs 3, 4, 7, 8, and 12 were contained in most *MdNF*-*YA* genes whereas *MdNF-YA9* and *MdNF-YA10* lacked motif 8. Most of the *MdNF*-*YB* genes contained motif 1, 2, and 3 except *MdNF*-*YB4, MdNF-YB14*, and *MdNF*-*YB17*. Most of the *MdNF*-*YC* genes contained seven types of motifs (motif 1, 4, 5, 6, 9, 10, and 14). However, *MdNF*-*YC3* and *MdNF*-*YC9* presented different motif types compared with the other *MdNF*-*YC* genes. In addition, the gene structures of the various *MdNF-Y* subunits also displayed differences in the distribution of exons and introns, whereas the gene structures within each *MdNF-Y* subunit were relatively similar (Figure 2B). For example, in the *MdNF-YA* subfamily, the CDS were separated by four introns except *MdNF*-*YA4*, and all *MdNF*-*YC*s contained a long CDS region which is not separated by introns. 

### 2.3. Chromosome Distribution and Synteny Analysis of MdNF-Y Family Genes

**Figure 3:**
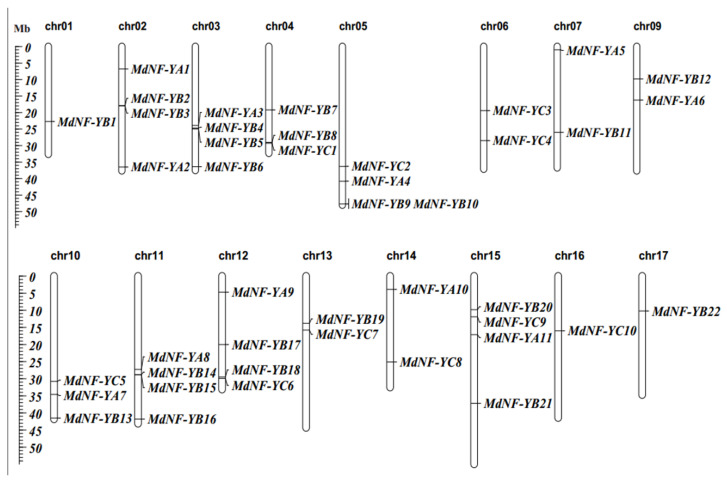
Physical distribution and chromosomal location of 43 *MdNF-Ys* genes in apple genome. 43 *MdNF-Ys* genes were mapped onto 16 chromosomes. Hollow bars indicated the chromosomes. Chromosomes and *MdNF*-*Ys* genes names were shown at the top and right of the bar, respectively. Scale bars located on left side of figure representing the length of each chromosome are in megabases (Mb).

To understand the chromosomal distribution of the different *MdNF-Ys*, chromosomal location map was created (Figure 3). All *MdNF-Y* genes were unevenly distributed on 16 of the 17 apple chromosomes except chr08. The number of genes on the chromosomes that contained *MdNF-Y* genes varied from one to four. Six chromosomes with four *MdNF-Y* genes, including chr02, chr03, chr05, chr11, chr12, and chr15, while only *MdNF-YB1* was located on the chr01. In addition, two *MdNF-Ys* were located on chr06, chr07, chr09, chr13, and chr14, and three *MdNF-Ys* were located on the terminus of chr04 and chr10, respectively. Distribution of *MdNF-Ys* was concentrated on the terminus of several chromosomes including chr03, chr04, chr05, chr10, and chr11.

**Figure 4:**
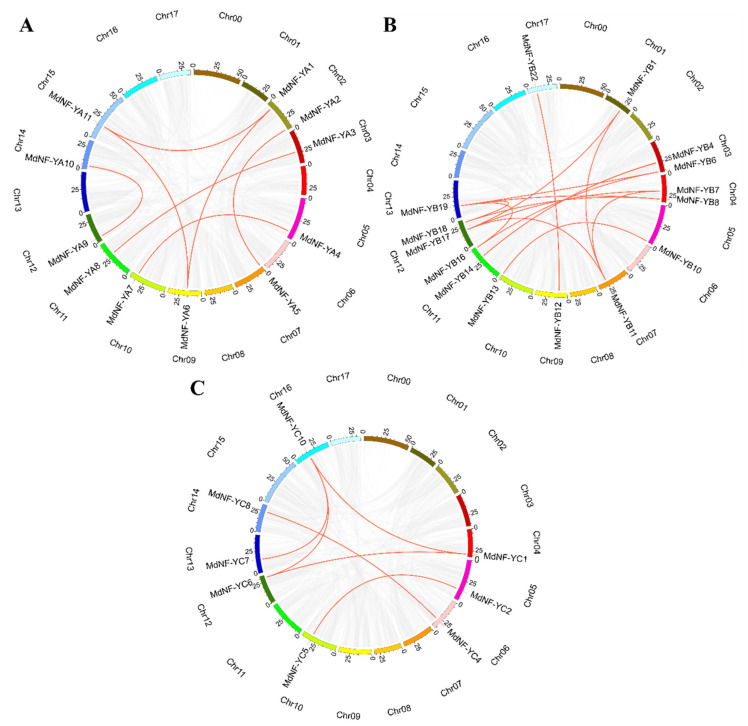
Synteny analysis of *MdNF-YAs* (**A**), *MdNF-YBs* (**B**) and *MdNF-YCs* (**C**) in the apple. The chromosomal localizations were shown for apple was random colors (chr01-17). The red lines indicated the segmental duplication. Gray lines represented all synteny blocks in the apple genome.

To investigate the gene family expansion mechanism of the *MdNF-Y* genes, we analyzed their synteny relationships in apple genomes (Figure 4). The results showed that no tandem duplication event had occurred but that 27 pairs of gene segmental duplication events could be identified (Figure 4, Table S2). Overall, 11 *MdNF-YA*, 15 *MdNF-YB*, and 8 *MdNF-YC* genes were mapped to the 16 chromosomes except for chr 08, while the pairs of paralogous genes were 7, 14, and 6, respectively. These results suggested that segmental duplications were the cause of *MdNF-Y* genes amplification. Interestingly, we found that there was respectively one triangular relationship in the *MdNF*-*YA* (*MdNF*-*YA1, MdNF*-*YA6,* and *MdNF*-*YA11*) and *MdNF-YC* subunits (*MdNF-YC1, MdNF-YC6,* and *MdNF-YC10*). There were four triangular relationships in *MdNF-YB* subunit including *MdNF-YB1, MdNF-YB11,* and *MdNF-YB17*; *MdNF-YB6*, *MdNF-YB16,* and *MdNF-YB19*; *MdNF-YB7*, *MdNF-YB11,* and *MdNF-YB17*; and *MdNF-YB8*, *MdNF-YB18,* and *MdNF-YB19*. In addition, more gene pairs are listed in Table S2.

**Figure 5:**
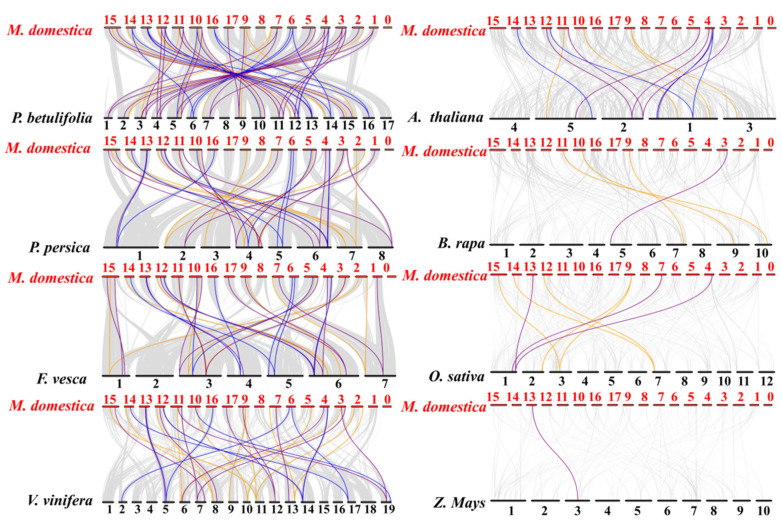
Synteny analysis of *MdNF-Ys* with other eight species, including *P. betulifolia* (pear), *P. persica* (peach), *F. vesca* (strawberry), *V. vinifera*(grape), *A. thaliana* (Arabidopsis), *O. sativa* (rice), *Z. Mays* (maize), and *B. rapa* (Chinese cabbage). The red solid bars represent the chromosomes of apple, while the black solid bars represent the chromosomes of other species. The gray lines represent the collinear blocks within apple and other plant genomes, while the yellow, purple, and blue lines highlight syntenic *MdNF*-*YA, MdNF*-*YB,* and *MdNF*-*YC* gene pairs, respectively.

To further study the gene expansion relationship and evolution of *NF*-*Y* subunit*s* (Figure 5), we chose eight representative plant models with widely ranging homologies to analyze the synteny of *NF*-*Y* genes. The eight species contained six dicots, including three Rosacea species (*Pyrus* *betulifolia*, *Prunus persica,* and *Fragaria vesca*), *Vitis vinifera*, *A. thaliana,* and *Brassica rapa* and two monocots (*Oryza sativa L.* and *Zea Mays L*). The results suggested that many *NF*-*Y* genes in apple have homology to reference plants. It is well known that apples also belong to the Rosaceae, like *F. vesca*, *P. betulifolia*, and *P. persica*. Furtherly, many ortholog pairs of *MdNF-Y* genes were found among *P. betulifolia* (59 orthologous gene pairs distributed on all apple chr except chr8), *P. persica* (37 orthologous gene pairs distributed on all apple chr except chr8 and chr17), and *F. vesca* (37 orthologous gene pairs distributed on all apple chr except chr8), respectively. Further, with the exception of *MdNF*-*YA1* and *MdNF*-*YB10,* the majority of *MdNF*-*Y* subunits had orthologous pairs in pears, which indicated that the NF-Y transcription factor families are highly homologous in apples and pears. However, fewer homologous gene pairs were observed between apple and *O. sativa L. (*only seven) and between apple and *Z. Mays. (*only one) (Table S3). In addition, we found some highly homologous genes were preserved during the species evolution. For example, *MdNF*-*YA6* had homologous pairs in seven species excepted maize, and *MdNF*-*YB19* had homologous pairs in six species excepted *Arabidopsis* and *B. rapa*. Likewise, *MdNF*-*YC1*, *MdNF*-*YC6,* and *MdNF*-*YC8* had homologous pairs in five species, respectively.

### 2.4. The Cis-Acting Regulatory Members in the Promoter of MdNF-Y Family Genes

**Figure 6:**
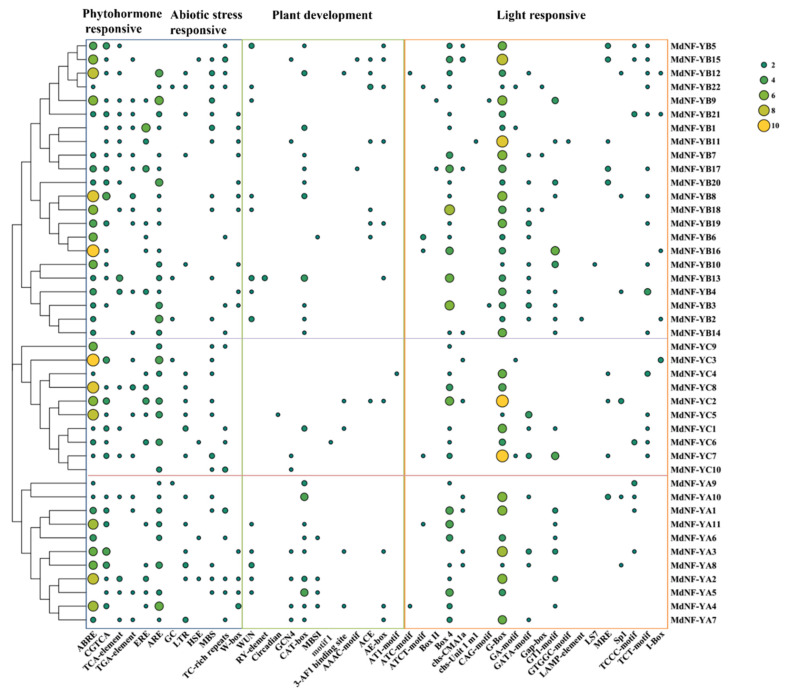
The cis-acting regulatory elements of promoters in apple *MdNF-Ys* genes. The blue, green, and orange blocks represent phytohormone and abiotic stress, plant development, and light responsive cis-elements, respectively. The number of cis-acting elements was indicated by different colors and circle sizes. The size of green to yellow circle represented the number of cis-acting elements.

To predict the potential function of *MdNF*-*Y* transcription factors, we chose the 1500 bp upstream sequences using plantCARE to analyze the type of cis-elements in the promoter (Figure 6 and Table S5). We found a preponderant number of TATA-boxes and CAAT-boxes, which have been analyzed in terms of their roles in transcription [38]. The results also revealed the presence of a very large number of light cis-elements. Therefore, we classified the results into four broad categories: Those participated in plant development, phytohormones, abiotic, and biotic stress-responsive, and light-responsive. The cis-elements participated in light responsiveness included a 3-AF1 binding site, G-Box, GA-motif, and a GATA-motif. The cis-elements involved in plant development included flavonoid biosynthetic genes regulation (MBSI), endosperm expression (GCN4), meristem expression (CAT-box), circadian control (Circadian), Seed (RY-element), and root-specific (motif I). The cis-elements partake in abiotic and biotic stress included anaerobic induction (ARE), heat stress responsiveness (HSE), low-temperature responsiveness (LTR), anoxic specific inducibility (GC), drought-inducibility (MBS), defense and stress responsiveness (TC-rich repeats), auxin-responsive element (TGA-element), pathogen (W-box), and wound-responsive element (WUN-motif). The cis-elements involved in phytohormone responsive included abscisic acid (ABA) responsiveness (ABRE) MeJA-responsiveness (CGTCA-motif), ethylene (ERE), and salicylic acid (SA) responsiveness (TCA-element).

In addition, most of the *MdNF*-*Ys* had ABRE cis-acting regulatory elements with a total of 169 elements, indicating that *MdNF*-*Y* genes play an important role in ABA response. All *MdNF*-*Y* genes contained more or less different types of light-responsive elements, although all *MdNF*-*YBs* possessed a G-Box element. A number of cis-acting regulatory elements involved in plant development were found in some of the *MdNF*-*Y* genes. For example, motif I, which was involved in plant root development [39], was only found in *MdNF*-*YC6*. A circadian cis-acting element, involved in circadian control [40], was only discovered in *MdNF*-*YC4*. An RY-element, participated in plant seed-specific regulation [41], was only detected in *MdNF*-*YB13*. Therefore, these *MdNF*-*Y* genes need to be further explored since they may play a critical role in apple growth. The specific cis-element analysis of the *MdNF*-*Y* genes is shown in Table S5. Actually, some reports in other plants have indicated that *NF-Ys* participated in response to abiotic stresses (cold [42], heat [25], drought [43], and salinity [28], root development [44], seed-specific regulation [44] and photoperiod-dependent flowering [29]. So, we further detected the expression levels of *MdNF*-*Y* genes in different tissues and under various abiotic stresses in subsequent experiments.

### 2.5. Protein Interaction Analysis of MdNF-Y Genes

**Figure 7:**
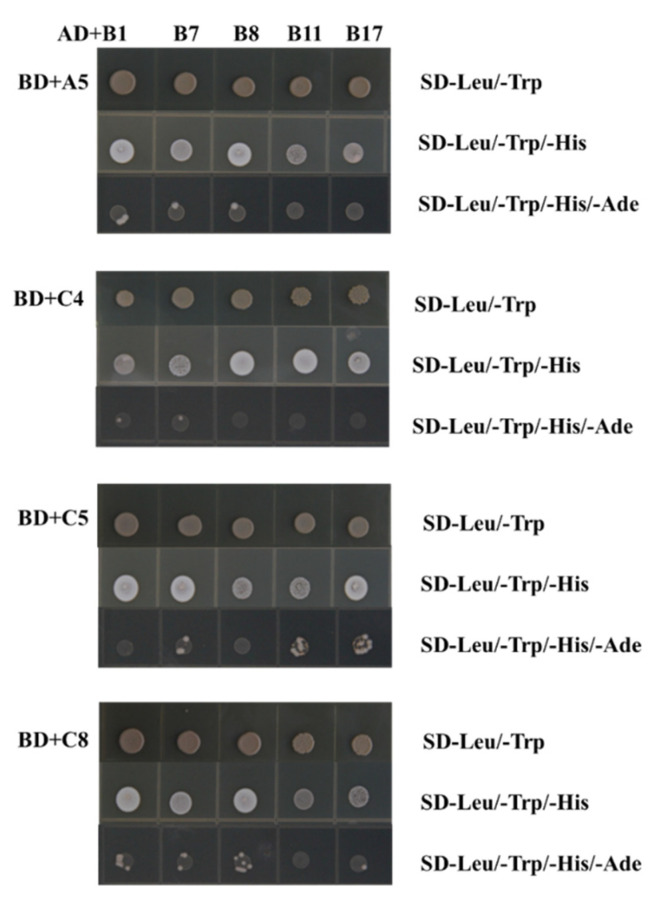
Protein–protein interactions between MdNF-Y subunits by yeast two hybrid. *MdNF*-*YB* genes were constructed to vectors with GAL4 activation domain (AD), *MdNF*-*YA* and *MdNF*-*YC* genes with GAL4 DNA-binding domain (BD).

NF-YB, which does not contain a nuclear localization signal, needs to form a tight dimer with NF-YC in order to translocate from the cytoplasm to the nucleus, and subsequently binds to NF-YA to form a heterotrimeric complex. Then, the complex can interact with other regulatory factors to activate or repress the expression of downstream genes [45,46]. To determine the potential interactions between MdNF-YB and MdNF-YC, or between MdNF-YB and MdNF-YA members, we detected a physical interaction of some *MdNF*-*Y* gene products including five MdNF-YBs, three MdNF-YCs, and one MdNF-YA in the Y2H system (Figure 7). The full CDS of five *MdNF*-*YB*s were fused with the activation domain (AD) of the pGADT7 vector (expressing the “prey”), while the CDS of three *MdNF*-*YCs* and one *MdNF*-*YA* were fused to the DNA-binding domain (BD) of the pGBKT7 vector (expressing the “bait”). After pairwise co-transforming AD- and BD-expressing vectors into the Y2H Gold yeast strain, almost all yeast cells bearing both the *MdNF*-*YB* and *MdNF*-*YC*-expressing plasmids, or both *MdNF*-*YB* and *MdNF*-*YA*-expressing plasmids (except yeast cells bearing both *MdNF*-*YB11* and *MdNF*-*YA5*) were capable of growth on SD-Leu/-Trp/-His medium indicating an interaction between prey and bait. Then, the surviving yeast cells were transferred to SD-Leu/-Trp/-His/-Ade medium. *MdNF*-*YC5* showed strong interaction signals with *MdNF*-*YB7, 11,* and *17. MdNF*-*YC8* showed strong interaction signals with *MdNF*-*YB1* and *MdNF*-*YB8. MdNF*-*YB1* showed weak interaction signals with *MdNF*-*YA5* and *MdNF*-*YC4,* and *5. MdNF*-*YC4* showed no interaction signals with *MdNF*-*YB8, 11, or 17. MdNF*-*YA5* showed no interaction signals with *MdNF*-*YB11 and 17.* Taken together, these results suggested that many *MdNF*-*YB*s could interact with *MdNF*-*YAs* or three *MdNF*-*YC* genes on SD-Leu/-Trp/-His/-Ade medium.

### 2.6. Transcript Profiles Analysis of MdNF-Y Family Genes in Different Apple Tissues

**Figure 8:**
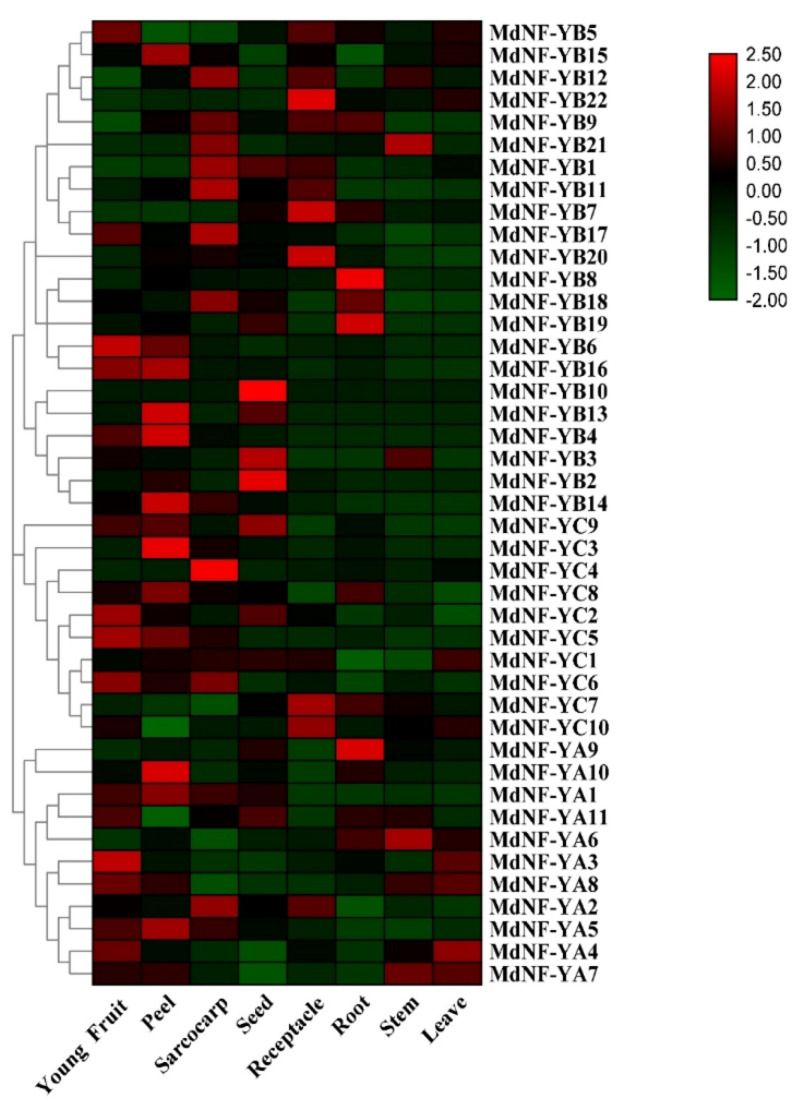
Expression profiles of *MdNF*-*Ys* in various apple tissues including roots, stems, leaves, receptacles, sarcocarps, young fruits, and seeds. In the heat map, values were transformed to log2 (value). Green, low expression; black, medium expression; red, high expression. *MdActin* was used as an internal control. The results were based on three biological replicates and three technical replicates.

To initially understand the function of the apple *MdNF-Y* genes, we analyzed the transcript profiles of the *MdNF-Y* gene family in roots, stems, leaves, receptacles, peel, sarcocarps, young fruits, and seeds using qRT-PCR. In general, the 43 *MdNF-Y* genes show diverse tissue-specific expression patterns and spatiotemporal expression characteristics (Figure 8). For the MdNF-YA subfamily, many genes had higher expression levels in vegetative and reproductive organs. For example, *MdNF*-*YA3, 4*, *7, 8* were strongly expressed in both young fruit and leaves. Moreover, *MdNF-YA6* was highly expressed in roots, stem, and leaves, and *MdNF-YA9* were highly expressed in roots. For the MdNF-YB subfamily, *MdNF*-*YB1*, *9,11,12,17,18* was highly expressed in sarcocarps, and *MdNF*-*YB2, 4, 6, 13, 14, 16* were highly expressed in peel. *MdNF*-*YB8*, *18, 19* were highly expressed in root tissue. In addition, all *MdNF-YC* genes had higher expression levels in at least one reproductive organ, such as receptacles, peel, sarcocarps, young fruits, and seeds. However, it is worth noting that *MdNF-Y* genes sharing very high sequence and exon–intron structure similarity in duplicated genomic regions (Figure 2), exhibited similar expression patterns (Figure 8). For example, *MdNF*-*YB1*, *MdNF*-*YB11,* and *MdNF-YB17* located in the duplicated genomic regions, were all highly expressed in sarcocarp. On the other hand, *MdNF*-*YB8*, *MdNF*-*YB18,* and *MdNF-YB19* were highly expressed in root tissue. Overall, the overlapping but distinct expression patterns of *MdNF*-*Y* genes indicated that the MdNF-Y family plays a critical role in different growth and development stages of apples.

### 2.7. Expression Levels of MdNF-Y Genes under Different Abiotic Stresses

**Figure 9:**
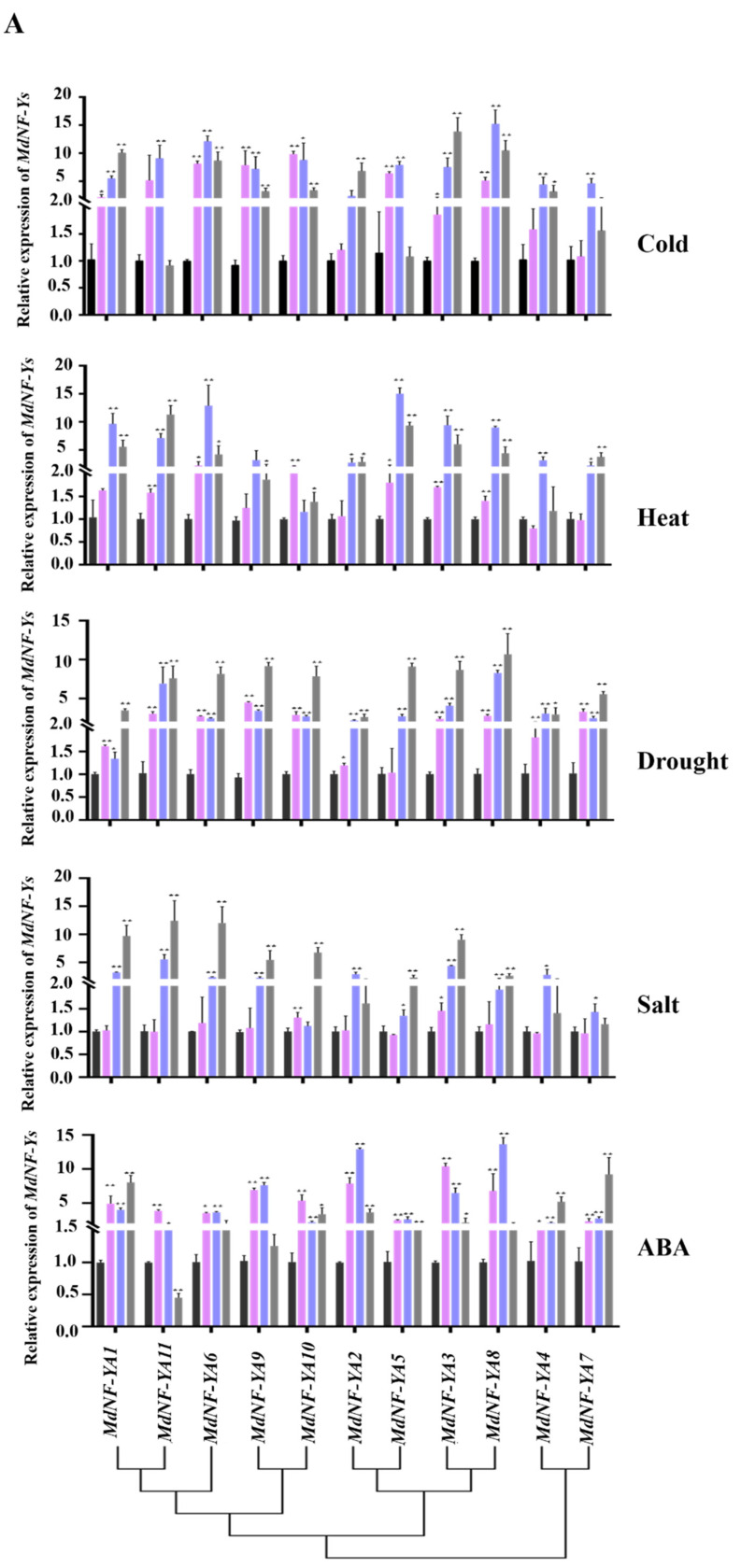

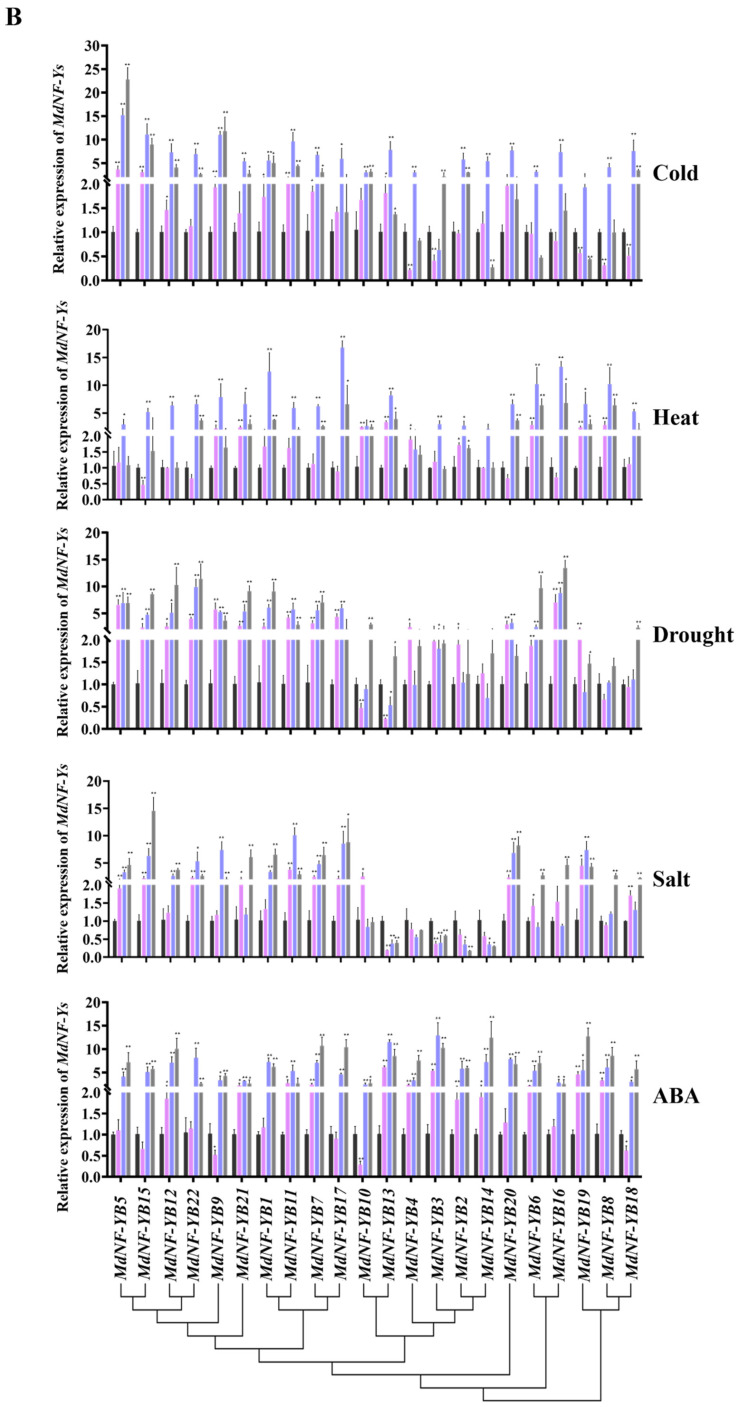

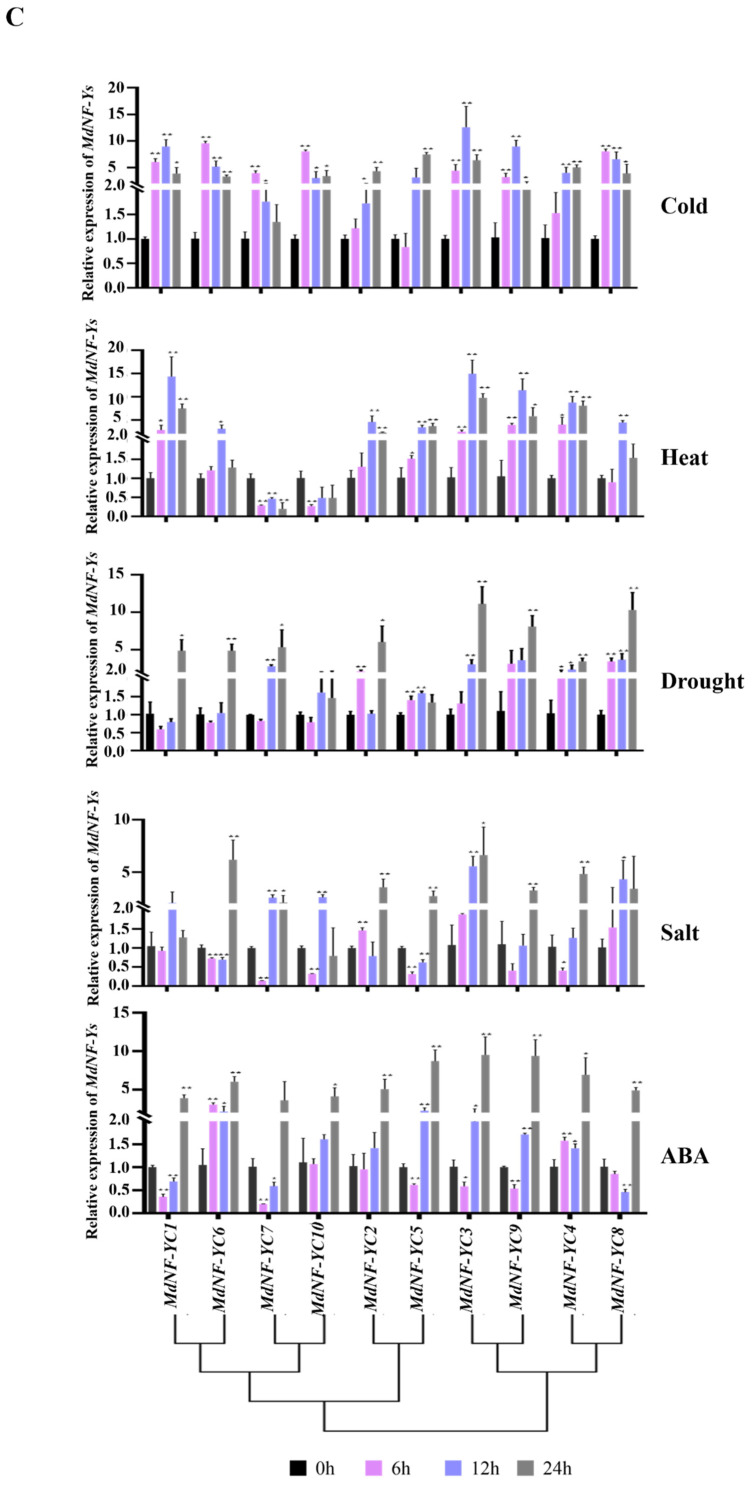
Expression profiles of *MdNF-YAs* (**A**), *MdNF-YBs* (**B**) and *MdNF-YCs* (**C**) in response to abiotic stress treatment, including ABA, drought, salinity, heat, and cold. The black, pink, purple, and gray bars represented the abiotic stress treatment time from 0, 6, 12, to 24 h, respectively. The black lines indicated error bars. The expression of *MdNF-Ys* at 0 h were set to 1 and *MdActin* was used as an internal control. Asterisks denote significance determined by *t*-test: * *p* < 0.05 and ** *p* < 0.01. The results were based on three biological replicates and three technical replicates.

Previous studies have indicated that NF-Y not only regulates plant growing development but also responds to abiotic stresses [47]. Similarly, in our study, a very high number of abiotic and biotic stress-responsive elements were detected in the upstream promoter of *MdNF-Y* genes (Figure 6). Therefore, we used qRT-PCR to investigate the response of the *MdNF*-*Y* gene family to abiotic stress. The transcriptional profiles of *MdNF*-*Y* genes under abiotic stresses from 0 to 24 h were monitored in this study (Figure 9). Under low-temperature treatment, the expression of all *MdNF*-*YAs* and *MdNF*-*YCs* was upregulated and the expression levels of *MdNF*-*YA* genes were higher than those of *MdNF*-*YC* genes as a whole (Figure 9). In *MdNF*-*YB* gene family, *MdNF*-*YB3*, *MdNF*-*YB5,* and *MdNF*-*YB9* gene expression levels reached their peak at 24 h whereas the expression of the rest of the *MdNF*-*YB* genes reached their peak at 12 h. The expression of *MdNF*-*YB3* decreased at first but had recovered at 24 h. Under high-temperature treatment, the expression levels of most *MdNF*-*Y* genes were upregulated except *MdNF*-*YC7* and *MdNF*-*YC10* that were downregulated (Figure 9C). Interestingly, of the *MdNF*-*YA* subfamily, *MdNF*-*YA3, MdNF*-*YA5,* and *MdNF*-*YA8* were highly expressed under low- or high-temperature treatment. Under drought treatment, the *MdNF*-*Y* gene family members were all upregulated showing a strong response to water deficit (Figure 9). In *MdNF*-*YB* gene family, *MdNF*-*YB10* and *MdNF*-*YB13* genes were first downregulated after PEG treatment and recovered at 24 h post-treatment. Under salinity treatment, the expression levels of *MdNF*-*YB2*, *MdNF*-*YB3*, *MdNF*-*YB4*, *MdNF*-*YB13,* and *MdNF*-*YB14* were extremely low (Figure 9B). Under ABA treatment, the expression levels of all *MdNF*-*YAs* were upregulated (Figure 9A). Different *MdNF*-*YA* genes reached their peak at different post-treatment times, including *MdNF*-*YA3* at 6 h, *MdNF*-*YA1*, *MdNF*-*YA4,* and *MdNF*-*YA7* at 24 h, and the rest of *MdNF*-*YA* genes at 12 h. The expression levels of *MdNF*-*YB9*, *MdNF*-*YB10*, *MdNF*-*YB15*, *MdNF*-*YB17,* and *MdNF*-*YB18* were decreased at 6 h post-treatment but had recovered at 12 h post-treatment, while the rest of *MdNF*-*YB* genes was upregulated. The expression of 59% of the *MdNF*-*YB* genes reached their peak at 24 h. However, the expression of all *MdNF*-*YC* genes reached its peak at 24 h. With the exception of the *MdNF*-*YC4* and *MdNF*-*YC6* genes, the expression of other genes was first downregulated but then upregulated.

## 3. Discussion

### 3.1. Conservation, Evolutionary and Divergence of the MdNF-Y Gene Family in Apple

Studies on the *NF-Y* genes in plant species have been accumulating since the function and regulatory mechanism of the first plant *NF-Y* gene was identified [47,48,49]. To date, *NF-Y* genes have been identified from simple model plants to more complex plants, and from monocotyledons to dicotyledons [2,15,16,50,51], such as Arabidopsis, maize, rice, grapes, rubber trees, and so on. However, there is few reports concerning the *NF-Y* gene family in apple. The number of *NF-Y* genes identified from plants varied from 13 in tomato [52] to 50 in maize [53]. In this study, we first systematically identified and analyzed the *NF-Y* gene family in apple at the genomic level and discovered 43 *MdNF-Y* genes in the apple genome, which was a greater number than for most of the other plants investigated so far.

Duplications at gene, chromosomal, or entire genomic level have been considered a major source of evolution, contributing to the origin of new gene functions and expression patterns [54]. Therefore, we further identified 27 paralogous pairs from segmental duplication events in apple, including 7 *MdNF-YAs,* 14 *MdNF-YBs*, and 6 *MdNF-YCs* (Figure 4 and Table S2). However, only 11 or 12 paralogous segmental duplication events had happened in *S. bicolor L.* or maize, while a total of 42 and 50 NF-Y proteins were respectively identified in those species [53,55]. Moreover, the genome size of plant NF-Y members studied greatly varied, from 265 Mb in peach to 742 Mb in apple, and only five pairs of paralogous events were found in peach [16]. These results all suggested that a potential correlation between *MdNF-Y* gene duplications and genome expansion existed during the species evolution.

In addition, the majority *NF-Y* genes from *M. domestica* were located in syntenic regions of other eight species genomes (*P. betulifolia*, *P. persica*, *F. vesca*, *V. vinifera*, *A. thaliana*, *B. rapa*, *O. sativa* and *Z. Mays*) (Figure 5 and Table S3). From the evolutionary data on *NF-Y* genes, we found that the number of ortholog pairs of apple and other species were related to their evolutionary relationship. It is widely known that *M. domestica*, *F. vesca*, *P. betulifolia*, and *P. persica* all belong to the Rosaceae and have a closer relationship than other species selected. As expected, they have significantly more ortholog pairs with apples than other species. Therefore, we concluded that the conservation of gene duplication during species evolution also supports the great differentiation of genome evolution. Meanwhile, the ortholog pairs from other plants can provide references to determine the function and mechanisms of apple NF-Y transcription factors.

Gene structural analysis of *MdNF-Y* subfamilies showed that there were many similarities in each subfamily with the corresponding subfamily in other species. For example, the genes in the *MdNF-YA* subunit, were interrupted by at least four introns. While, many *MdNF-YB* genes lacked introns. Intriguingly, *MdNF-YB17* and *CsNF-YB16*, as the longest apple or tea *NF-YB* gene, both have five introns [56]. The result was consistent with the gene structure of *PpNF-Y* and *CsNF-Y* families [14,16].

### 3.2. Differentially Expression Pattern of MdNF-Y Genes in Apple Tissues

To date, *NF-Y* genes have been found to play critical roles in regulating flower and fruit development, as well as various other physiological processes; however, their roles in apple have remained unclear. Therefore, in this study, we predicted the functions of the apple *NF-Y* genes based on their other species ortholog pairs in syntenic regions of the two genomes (Figure 5).

In general, the *NF-Y* genes exhibited distinct spatiotemporal expression patterns in apple tissues and organs. However, *NF-Y* genes with very high sequence and exon-intron structure similarity have similar expression patterns and gene function in different apple tissues. For example, *MdNF-YB1* and *MdNF-YB11*, located in the duplicated genomic regions, were all highly expressed in the sarcocarp and receptacle (Figure 4 and Figure 8). Apple fruits are considered ’false fruits’, since the sarcocarp has developed from the receptacle. Therefore, we hypothesized that these three homologous genes may play a synergistic role in the development of the receptacle into the fruit. In addition, the interactions between MdNF-YB17 and MdNF-YC5 or MdNF-YC8 proteins were identified by the Y2H experiments (Figure 7). Interestingly, *MdNF-YC5* and *MdNF-YC8* were also highly expressed in the sarcocarp or peel (Figure 8), indicating that they may play an important role in apple reproductive growth. Actually, many studies of NF-Ys in other plants have supported this hypothesis. In Arabidopsis, NF-YB and NF-YC subunits could interact with CONSTANS (CO) to form complexes, and further affected FLOWERING LOCUS T (FT) expression to induce the floral transition [2,23,57]. Analogously, *ZmNF-YA3*, in complex with CO and flowering promoting factor1 (FPF1) could bind to the FT-like12 promoter to promote early flowering in maize [29]. In addition, overexpression of the *TaNF-YB4* gene significantly improved transgenic wheat grain yield [58].

LEAFY COTYLEDON1 (LEC1), also known as *NF-YB9* is a key regulator that controls the complex process of seed development in Arabidopsis [44]. *AtNF-YB9* (LEC1) and *AtNF-YB6* (LEC1-Like) were both expressed in seeds [59]. Previous research has suggested that the gene expression patterns are related to the complex process of seed development which is highly coordinated both temporally and spatially in cellular processes [60]. In this study, we found that *MdNF-YB13* gene promoter contained an RY-element, which is involved in plant seed-specific regulation (Figure 6). Interestingly, *MdNF-YB13* and *MdNF-YB10*, located in the duplicated genomic regions, are ortholog genes of *AtNF-YB6* (LEC1-Like) and *AtNF-YB9* (LEC1) (Figureas 4 and 5 and Table S3), and also show high expression levels in seeds compared with other tissues (Figure 8). Recent studies have demonstrated that LEC1 combinates with other TFs, such as ABA-RESPONSIVE ELEMENT BINDING PROTEIN3 (AREB3), bZIP67, and ABI3 to regulate the diverse stages of seed development [61]. Moreover, previous studies have shown that LEC1 acts as a regulon to regulate hormone synthesis genes and as an integrator for light- and hormone signals to play an important role in the development of plant embryo [62]. The result of cis-acting element analysis also showed that the promoter region of *MdNF-YB13* contained those important plant endogenous hormone and light elements during seed development, such as ABRE (ABA), TGA-element (auxin), and G-box (light). Taken together, our study provides a direction for future research of the analysis of the specific regulatory mechanism of *MdNF-YB10* and *MdNF-YB13* during apple seed development.

### 3.3. Function of MdNF-Y Genes in Abiotic Stress

It has been reported that TFs, including MYB, WRKY, and NAC participated in the fight against abiotic stress to help plants resist or optimize the changes in the environment [63,64,65,66]. To date, numerous studies have demonstrated that *NF-Y* genes also played an important role in abiotic stress response. For instance, overexpression of *AtNF-YB2* and *AtNF-YB3* in Arabidopsis specifically conferred tolerance to drought and heat stress, respectively [25]. Allogenic overexpression of the *CdtNF-YC1* transcription factor from bermudagrass enhanced the transgenic rice tolerance under the drought and salt treatment, through ABA-dependent and ABA-independent pathways [28]. Another recent study has shown that *PdNF-YB21* positively regulates the tolerance to drought stress by ABA-mediated IAA transport in Populus [30].

In addition, it is widely known that root is the major organ to respond to osmotic stress caused by drought and salt [43]. In this study, *MdNF-YA6*, *MdNF-YA9*, *MdNF-YB8*, *MdNF-YB9*, *MdNF-YB18*, *MdNF-YB19*, *MdNF-YC7*, and *MdNF-YC8* are mainly or specifically expressed in roots (Figure 8). Further, we found the promoter region of *MdNF-YB8*, *MdNF-YB9*, *MdNF-YB18*, and *MdNF-YC7* contains drought cis-acting element MBS (CAACTG), and *MdNF-YA6* contains defence cis-element TC-rich repeats (ATTCTCTAAC/GTTTTCTTAC) (Figure 6). Meanwhile, the RT-PCR results also showed that these genes were highly expressed under drought and salinity stress (Figure 9). Therefore, we hypothesized that they may response to abiotic stress by regulating root growth. Existing research has also verified this hypothesis. For example, overexpression of the transcription factor *AtNF-YB3* increased the length of primary root [67], and improved drought and heat tolerance in *A. thaliana* [25]. Overexpression of *PdNF-YB21* in poplar promoted root growth with highly lignified and enlarged xylem vessels, resulting in increased drought resistance [30]. *TaNF-YB4* plays an important role in root development and in nitrogen and phosphorus uptake in wheat [58]. Higher levels *CdtNF-YC1* was detected in roots of bermudagrass and overexpression of *CdtNF-YC1* elevated tolerance to drought and salt stress in transgenic rice [28].

In summary, the *MdNF*-*Y* genes displayed different degrees of responses to abiotic stress. The specific mechanisms were studied in model plants. Although the detailed molecular mechanisms of the responses of *MdNF*-*Y* genes to abiotic stress remain unclear, this study points to a number of genes that deserve further exploration in future studies.

## 4. Materials and Methods

### 4.1. Plant Materials and Treatments

The apple Xinjiang No. 1 tissue culture seedlings used for stress treatment was cultured in a specific-medium with Murashigeand Skoog (MS) medium, 0.8% agar, 0.5 mmol L^−1^ indole-3-butytric acid (IBA), and 0.7 mmol L^−1^ 6-benzylaminopurine (6-BA). Apple tissue culture seedlings were first grown in specific-medium formulation under 16 h light/8 h dark at 25 °C environmental conditions for thirty days. Thirty days later, 10% polyethylene glycol (PEG) 6000, 100 mmol L^−1^ NaCl, and 100 mmol L^−1^ Abscisic acid (ABA) were added to the specific medium, respectively, to induce the stress response of apple tissue culture seedlings and cultivated under the above environmental conditions. Besides, apple tissue culture seedlings were cultured in a specific medium, and the environmental temperature was adjusted to 4 °C and 40 °C, respectively. After the above five stress treatments, samples of apple tissue culture seedlings were collected at 0, 6, 12, and 24 h respectively, frozen rapidly with liquid nitrogen, and stored in −80 °C refrigerator for succedent experiments [68]. For all treatments, three biological replicates were collected.

The different apple tissues, including root, stem, leaves, peel, receptacle, young fruits (20 days after flowering), sarcocarp, and seed was obtained from seven-year-old ‘Xinjiang No.1’ apple trees at the experimental station (longitude 120°39′ E, latitude 36°27′ N) of Qing Dao Agricultural University in 2018. These tissues materials were frozen rapidly with liquid nitrogen and stored in −80 °C refrigerator for subsequent experiments. For all sample, three biological replicates were collected.

### 4.2. Identification of Apple MdNF-Y Genes

The amino acid sequences of Arabidopsis 30 AtNF-Y family were obtained from the TAIR database (https://www.arabidopsis.org/). After that, The NF-Y domains were searched from BlastP (https://www.rosaceae.org/blast/protein/protein) in the NCBI database in the apple genome in the NCBI database. The Hidden Markov Models (HMM) profile of the conserved domains of NF-YA (PF02045) and NF-YB (PF00808) were downloaded from the Pfam database (http://pfam.xfam.org/). Then, they were used to search for protein sequences in the apple genome. Apple candidate NF-Y gene family members obtained by the above two methods. To ensure accuracy, SMART (http://smart.embl-heidelberg.de/) and CCD programs were used to verify the existence of conserved Pfam and complete NF-Y domains. The amino acid number, PI, and molecular weight of the identified NF-Y protein sequence was obtained from the ExPASy website (http://web.expasy.org/). The location of *NF-Y* gene on chromosome was determined by Gene Structure Display Server (GSDS, http://gsds.cbi.pku.edu.cn) [69].

### 4.3. Alignments, Synteny Analysis of MdNF-Ys

Multiple sequence alignments were performed on 43 *MdNF*-*Y* protein sequences by using DNAMAN 9 with its default. MCScanx (http://chibba.pgml.uga.edu/mcscan2/) software was used to search the homologous genes of *MdNF-Y* and their collinearity was obtained [70]. Circos (http://circos.ca/) software was used to analyze the collinearity of the *MdNF*-*Y* gene family [71].

### 4.4. Phylogenetic, Conversed Motifs, and Gene Structure Analysis of MdNF-Ys

The CDS and DNA sequences of *MdNF-Y* were obtained from the Apple Genome Browser. Using MEGA 7 software, the Neighbor-Joining (NJ) method was used to construct the phylogenetic tree of apple and Arabidopsis NF-Y protein sequences. Bootstrap was set to 1000 replicates. The protein sequence of the candidate *MdNF*-*Y* gene was analyzed using MEME (http://meme-suite.org/) software [72]. Set to look for 20 motifs. Phylogenetic tree, conserved motifs, and gene structure of apple *MdNF-Y* gene family was visualized on the TBtools toolkit [73].

### 4.5. Prediction of Cis-Acting Elements in Promoters of MdNF-Ys

The Plant CARE database was employed to predict the potential cis-acting elements in the 1500 bp promoters upstream region of the apple *MdNF*-*Ys* gene family [74], and visualized on the TBtools toolkit [73]. Details information for the promoters used were listed in Table S4.

### 4.6. Yeast Two Hybrid Assays

The CDS of *MdNF*-*YB*, *MdNF*-*YA,* or *MdNF*-*YC* genes were cloned into prey vector pGADT7 with the activating domain (AD). The CDS of *MdNF*-*YA* or *MdNF*-*YC* genes were cloned into bait vector pGBKT7 with the DNA binding domain (BD). The primers were listed in Table S6. Two constructs were co-transformed into the Y2H Gold yeast strain according to the protocol (www.weidibio.com). Then, these yeast strains were cultured on SD(-Leu/-Trp) growth medium (Clontech) and selected on SD(-Leu/-Trp/-His) and SD(-Leu/-Trp/-His/-Ade) screening medium (Clontech). 

### 4.7. Quantitative Real-Time RT-PCR Analysis

RNA from various stress treatments (ABA, drought, salinity, heat, and cold) or different apple tissues (root, stem, leaves, peel, receptacle, young fruits (20 days after flowering), sarcocarp, and seed) was extracted with the EASYspin Plant RNA Rapid Extraction Kit (YPHBIO, Beijing, China), and the extracted RNA concentration was determined by the instrument Nano Drop 2000 (Gene Company Limited, Hong Kong, China). The cDNA was obtained by using the reverse transcription kit (Takara, Dalian, China) according to the manufacturer’s methods. Quantitative real-time PCR (qRT-PCR) was performed with ChamQ SYBR Color qPCR Master Mix (Without ROX) (Vazyme, Nanjing, China) in Roche machine (Roche, Shanghai, China). The PCR program was as followed: 95 °C for 2 min, 40 cycles of 95 °C for 30 s, 56 °C for 30 s and 72 °C for 30 s with a final dissociation stage. The internal reference gene, *MdActin* (nucleotide, CN938023), was used to normalize the expression levels of the tested genes. The primers for qRT-PCR experiment were designed on NCBI-BLAST (https://blast.ncbi.nlm.nih.gov/Blast.cgi) and listed in Table S6. *MdActin* was used as an internal control. The relative expression of *MdNF-Y* genes was calculated with 2^−ΔΔCt^ methods [75] and figured by GraphPad.Prism.5.0 software. Three biological replicates with three technical replicates each were measured. Transcripts profiles from different apple tissues were visualized heat-mapped using the TBtools toolbox [73].

## 5. Conclusions

In this work, 43 *MdNF-Y* genes (11 *MdNF-YAs*, 22 *MdNF-YBs,* and 10 *MdNF-YCs*) were identified and their evolutionary, structure, biological function, and expression pattern were analyzed. Based on prediction and experimental data, *MdNF-Y*s might play an important role in apple development and response to five abiotic stress (ABA, drought, heat, cold, and salinity). Our findings will contribute a foundation for further study of the functional and regulatory mechanisms controlled by the *NF-Y* gene family in apple.

## Figures and Tables

**Table 1:** Information of the *MdNF-Ys* genes in apple.

Name	Gene	Chromosome Location (bp)	DNA (bp)	CDS Length	Protein (aa)	pI	MW(Da)
MdNF-YA1	MD02G1086200	6760401–6763923	3522	927	308	9.02	33,933.04
MdNF-YA2	MD02G1309800	36538789–36544223	5434	1002	333	8.52	36,822.80
MdNF-YA3	MD03G1174600	23867258–23872067	4809	1077	358	7.04	38,855.31
MdNF-YA4	MD05G1273300	40831929–40836805	4876	207	68	6.42	7449.24
MdNF-YA5	MD07G1011300	1023634–1029101	5467	981	326	8.41	35,657.67
MdNF-YA6	MD09G1186200	16163341–16168480	5139	897	298	9.43	32,857.83
MdNF-YA7	MD10G1253300	34584154–34588714	4560	627	208	8.16	22,824.18
MdNF-YA8	MD11G1192400	27332752–27339954	7202	1047	348	8.43	37,923.42
MdNF-YA9	MD12G1042100	4650199–4655169	4970	969	322	9.07	35,109.45
MdNF-YA10	MD14G1041300	3873166–3878129	4963	930	309	9.63	33,939.24
MdNF-YA11	MD15G1213400	17122823–17125801	2978	903	300	9.25	33,209.33
MdNF-YB1	MD01G1112400	22658861–22662399	3538	645	214	8.70	22,929.78
MdNF-YB2	MD02G1191900	17939911–17940645	734	735	244	5.25	27,017.25
MdNF-YB3	MD02G1192100	18023631–18025409	1778	1044	347	4.80	38,127.12
MdNF-YB4	MD03G1179600	24691097–24691663	566	567	188	4.80	20,686.4
MdNF-YB5	MD03G1183400	25025010–25025594	584	585	194	6.76	20,837.01
MdNF-YB6	MD03G1283700	36413956–36414516	560	561	186	7.00	20,947.32
MdNF-YB7	MD04G1104700	19216567–19220090	3523	525	174	7.05	18,780.78
MdNF-YB8	MD04G1203300	28953974–28954426	452	453	150	6.60	16,863.98
MdNF-YB9	MD05G1361000	47685520–47685846	326	327	108	5.11	11,910.38
MdNF-YB10	MD05G1361600	47696633–47698977	2344	876	291	6.96	31,267.54
MdNF-YB11	MD07G1180200	25950191–25953495	3304	648	215	8.71	23,159.02
MdNF-YB12	MD09G1126900	9795758–9796411	653	654	217	7.44	22,184.36
MdNF-YB13	MD10G1339000	41496215–41496999	784	744	247	7.35	27,349.57
MdNF-YB14	MD11G1199300	28769434–28769985	551	552	183	5.89	20,252.83
MdNF-YB15	MD11G1200400	28876837–28877409	572	573	190	7.43	20,088.35
MdNF-YB16	MD11G1302500	41788083–41788580	497	498	165	6.69	18,596.63
MdNF-YB17	MD12G1124800	19970949–19980773	9824	519	172	5.81	18,595.6
MdNF-YB18	MD12G1217100	29460289–29460741	452	453	150	7.84	16,794.94
MdNF-YB19	MD13G1170200	13815353–13815826	473	1374	457	6.60	17,505.45
MdNF-YB20	MD15G1134600	9778391–9779101	710	711	236	6.85	26,721.4
MdNF-YB21	MD15G1334200	37152321–37152947	626	627	208	7.46	23,010.4
MdNF-YB22	MD17G1117200	10167264–10168240	976	720	239	5.58	24,810.33
MdNF-YC1	MD04G1207500	29269117–29271406	2289	807	268	6.46	29,749.6
MdNF-YC2	MD05G1229600	36298931–36300862	1931	807	268	5.36	29,718.64
MdNF-YC3	MD06G1078500	19362575–19362940	365	366	121	7.78	13,368.54
MdNF-YC4	MD06G1141200	28495891–28498876	2985	720	239	5.15	25,832.07
MdNF-YC5	MD10G1208800	30806582–30809166	2584	834	277	6.11	31,174.24
MdNF-YC6	MD12G1221800	29861745–29864137	2392	789	262	6.05	29,217.11
MdNF-YC7	MD13G1185200	15766106–15768771	2665	756	251	6.19	28,006.56
MdNF-YC8	MD14G1156400	25090589–25093134	2545	717	238	6.05	2577.04
MdNF-YC9	MD15G1159400	11916470–11917171	701	702	233	5.40	26,518.35
MdNF-YC10	MD16G1185900	16034439–16037111	2672	738	245	6.19	27,400.74

## Data Availability

The data presented in this study are available in supplementary material S1 to S6.

## Supplementary Materials

Supplementary Materials can be found at https://www.mdpi.com/2223-7747/10/1/16/s1. Table S1: MdNF-Y protein sequences and CDS length. Table S2: The paralogous gene pairs of 43 MdNF-Ys. Table S3: The ortholog pairs of apples with eight species. Table S4: The 1500 bp promoter sequences of the 43 *MdNF-Ys*. Table S5: cis-element analysis of the 43 *MdNF-Ys* in the 1500 bp promoter sequences. Table S6: The primers used in this study.
